# GatorST: A Versatile Contrastive Meta‐Learning Framework for Spatial Transcriptomic Data Analysis

**DOI:** 10.1002/smtd.202600006

**Published:** 2026-04-08

**Authors:** Zhenhao Zhang, Yuxi Liu, Song Wang, Qin Ma, Qianqian Song, Jiang Bian

**Affiliations:** ^1^ Biostatistics and Health Data Science School of Medicine Indiana University Indianapolis Indiana USA; ^2^ Department of Health Outcomes & Biomedical Informatics College of Medicine University of Florida Gainesville Florida USA; ^3^ Department of Electrical and Computer Engineering University of Virginia Charlottesville Virginia USA; ^4^ Department of Biomedical Informatics College of Medicine The Ohio State University Columbus Ohio USA

**Keywords:** contrastive learning, graph neural networks, meta learning, spatial domain identification, spatial transcriptomics

## Abstract

Recent advances in spatial transcriptomics (ST) have revolutionized the understanding of cellular functions by providing gene expression profiles with rich spatial context. Effectively learning spatial representations is essential for downstream analyses and requires robust integration of spatial and transcriptomic information. Although existing methods show promise, they often fail to capture local (neighbor‐level) and global (tissue‐wide) contexts, and methods based on contrastive learning often rely on augmentation strategies that introduce noise and instability. GatorST, a novel and versatile framework, explicitly integrates graph‐based modeling with advanced meta‐learning strategies to generate spatially informed representations of ST data. Locally, a spot‐spot graph connects each node to its nearest neighbors, while two‐hop subgraphs capture fine‐grained spatial context. Globally, gene expression profiles are clustered to produce pseudo‐labels, providing weak supervision for representation learning. An episodic training strategy inspired by meta‐learning further enhances GatorST's ability to generalize to new spatial contexts, ensuring robust integration of local and global spatial information. Comprehensive comparisons with fifteen state‐of‐the‐art methods demonstrate that GatorST consistently outperforms existing approaches in identifying spatial domains, imputing gene expression, removing batch effects, and inferring spatial trajectories. By integrating local spatial topology with global gene expression patterns, GatorST provides biologically meaningful representations that advance key downstream analyses.

## Graphical abstract

GatorST learns spatially informed embeddings for spatial transcriptomics by coupling two‐hop subgraph graph convolutions with cluster‐aware pseudo‐label supervision and episodic contrastive meta‐learning. The framework captures neighbor‐level topology and tissue‐wide expression semantics without heavy augmentations, delivering robust representations that improve spatial domain detection, gene‐expression imputation, batch integration, and trajectory inference across diverse tissues and platforms.

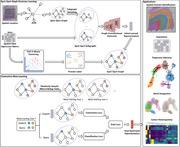

## 1 Introduction

Human organs and systems comprise a wide range of cell subpopulations, each contributing uniquely to physiological functions and processes. The spatial distribution of these cells and their interactions play a vital role in maintaining these functions [1]. Analyzing tissue regions and cells within their natural spatial context is therefore highly desirable. By examining the concordance and variability among tissue regions and cell types, we can gain a detailed understanding of intercellular communication, with significant implications for uncovering disease mechanisms [2, 3].

Recent advances in spatial transcriptomics (ST) [4] have provided gene expression profiles with spatial context, enabling unprecedented insights into cellular function, tissue organization, and microenvironmental interactions [5, 6, 7, 8, 9, 10, 11]. A key goal in ST analysis is to learn robust spatial representations that capture both gene expression and spatial architecture, as these representations are critical for downstream tasks such as identifying spatial domains, imputing gene expressions, removing batch effects, and inferring developmental trajectories [12]. However, achieving reliable and biologically meaningful representations is challenging due to the high dimensionality, sparsity, and technical noise inherent in ST data. Addressing these challenges requires computational methods that can effectively reduce dimensionality while preserving important spatial and biological signals.

Many recent methods have focused on clustering‐based strategies to identify spatial domains, with representative approaches including BayesSpace [13], UTAG [14], SpaGCN [15], SpaceFlow [16], and BANKSY [17]. These methods leverage various strategies such as probabilistic modeling, integration of spatial graphs, and deep graph learning to capture local spatial patterns. Graph‐based approaches (e.g., DeepST [18], CCST [19], STAGATE [20], Spatial‐MGCN [21], GraphST [22]) have been especially promising, using graph neural networks (GNNs) to integrate gene expression and spatial relationships for improved clustering accuracy. Some methods incorporate self‐supervised contrastive learning to enhance representation robustness. Despite these advances, most existing methods inadequately capture both local (neighbor‐level) and global (tissue‐wide) contexts simultaneously. Additionally, reliance on simplistic corruption or heavy augmentations can introduce noise and instability, particularly in small‐scale datasets, limiting the quality and interpretability of learned embeddings.

To overcome these limitations, we introduce GatorST, a novel framework tailored for spatial transcriptomics analysis. GatorST explicitly integrates local and global spatial contexts by constructing two‐hop neighborhood subgraphs that preserve fine‐grained spatial topology and by generating global pseudo‐labels via clustering of gene expression profiles as weak supervision. This design is incorporated into a contrastive learning framework that encourages spatial coherence and biological relevance in the learned embeddings. Furthermore, GatorST employs a meta‐learning‐inspired episodic training strategy to enhance generalization across diverse tissue types and spatial resolutions. Extensive evaluations across multiple datasets demonstrate that GatorST consistently outperforms state‐of‐the‐art methods in spatial domain identification and gene expression imputation, offering a scalable, robust, and generalizable solution for spatial transcriptomics analysis.

## 2 Results

### 2.1 Overview of GatorST Framework

**FIGURE 1:**
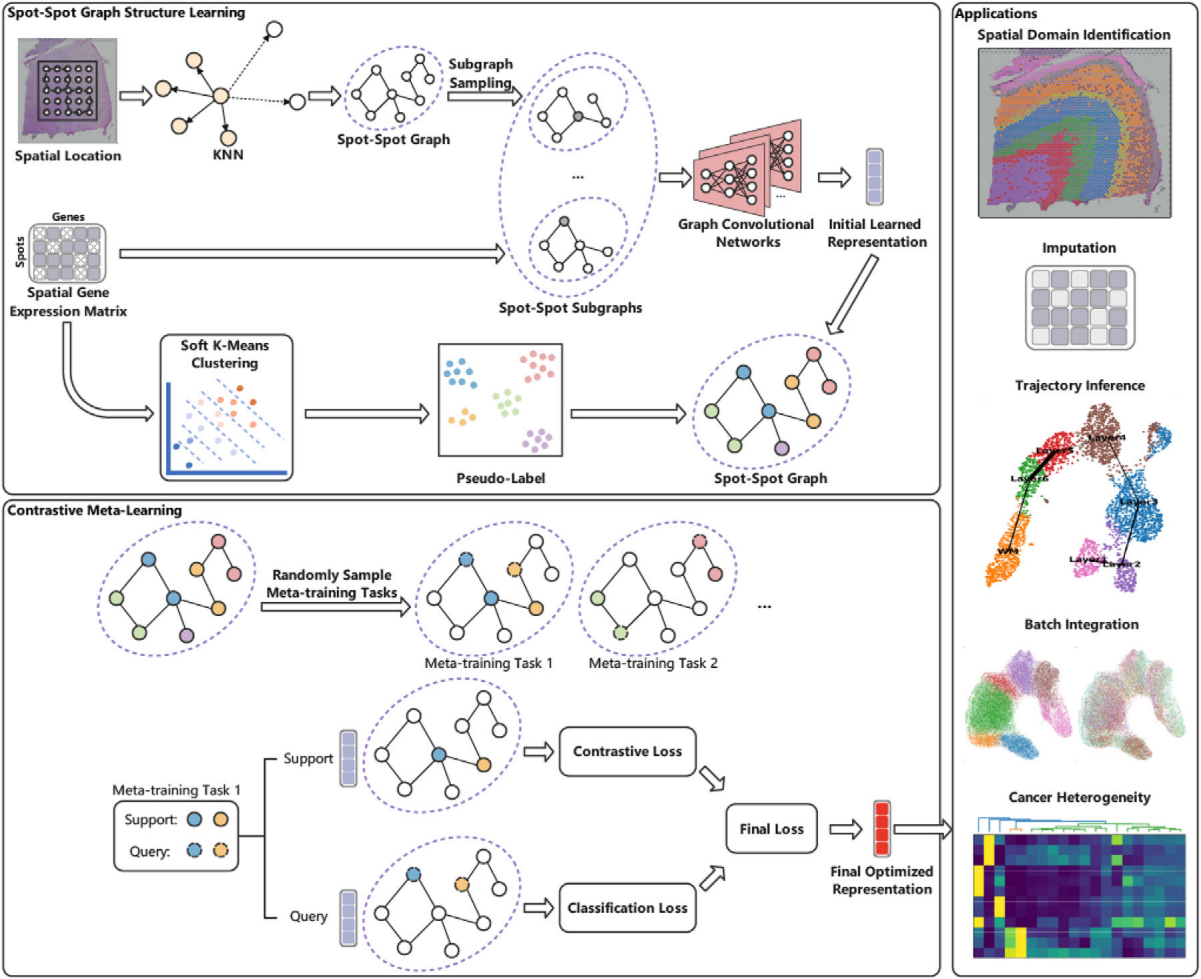
Overview of the GatorST framework.

GatorST is designed to learn robust and biologically meaningful representations of spatial transcriptomics data by integrating a graph‐based approach with contrastive meta‐learning, as shown in Figure 1. It begins by constructing a spot‐spot graph, where each node represents a spatial spot characterized by its gene expression profile and spatial coordinates. Edges are defined using a top‐k nearest neighbor strategy based on spatial proximity, enabling each node to capture local spatial context. To better model local structural relationships, a subgraph is extracted for each node, consisting of its two‐hop neighbors, thereby enriching local representations and providing a more nuanced understanding of cellular microenvironments. Graph Convolutional Networks (GCNs) are then applied to these subgraphs to derive initial spot‐level embeddings that integrate both gene expression and spatial structural information, serving as the basis for subsequent optimization.

Next, GatorST builds on these initial spot‐level embeddings by employing a contrastive meta‐learning approach to further refine and optimize them. Specifically, it first performs soft K‐means clustering on the gene expression profiles to assign each spot a pseudo‐label, providing weak supervision that promotes intra‐cluster cohesion and inter‐cluster separation. Leveraging these pseudo‐labels, the meta‐learning approach then applies an episodic training strategy, constructing meta‐training tasks by sampling support and query sets from the pseudo‐labeled data. In each episode, a two‐step optimization process is conducted: first, contrastive learning aligns embeddings within the same pseudo‐label group and separates those from different groups, explicitly leveraging subgraph‐based structural relationships. Second, a cross‐entropy classification loss is applied to the query set to further fine‐tune the embeddings and ensure adaptability to specific tasks. A combined objective function balances these two losses, resulting in final spot‐level embeddings that are both structurally coherent and semantically informative.

Finally, the optimized spot‐level embeddings demonstrate strong versatility and practical utility across various downstream tasks, including spatial domain identification, gene expression imputation, trajectory inference, and batch effect removal. These results highlight GatorST's potential as a flexible and powerful tool for comprehensive spatial transcriptomics analysis. Detailed implementation procedures are described in the Materials and Methods section.

### 2.2 GatorST Outperforms Benchmark Methods in Spatial Domain Identification

**FIGURE 2:**
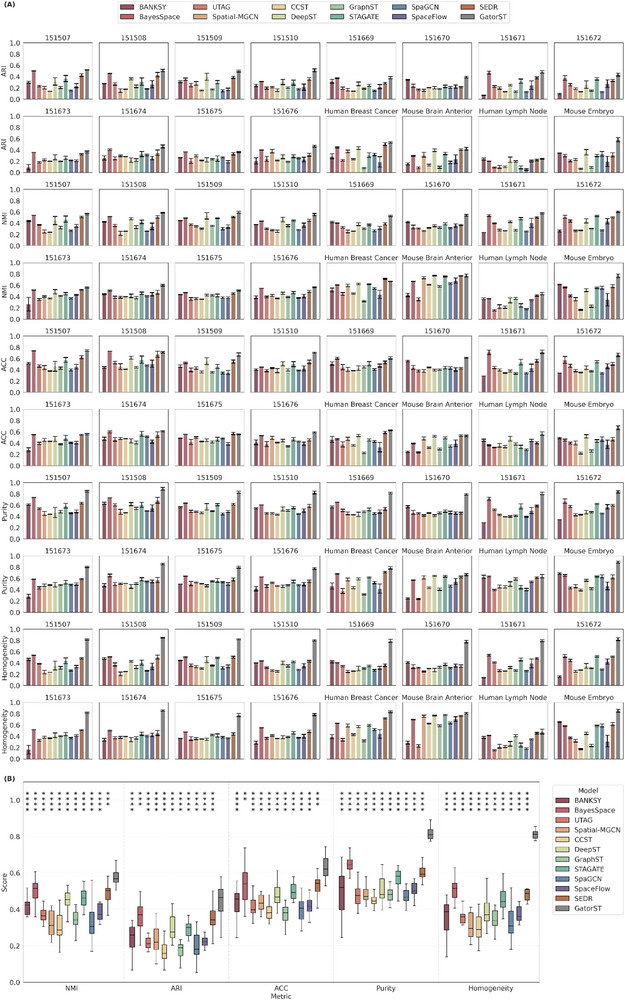
Clustering performance comparison across 16 spatial transcriptomic datasets. (A) For each dataset and method, ARI, NMI, ACC, Purity, and Homogeneity are shown as mean ± s.d. over 10 runs with different random seeds. (B) Boxplots summarize the distribution of dataset‐level mean scores from panel A across the 16 datasets. Statistical significance was assessed using pairwise two‐sided Wilcoxon signed‐rank tests comparing GatorST with each baseline on the paired dataset‐level mean scores. Higher values indicate better performance for all five metrics. Significance is denoted as n.s., adjusted *p* ≥ 0.05; ^*^ adjusted *p* < 0.05; ^**^ adjusted *p* < 0.005; ^***^ adjusted *p* < 0.0005; ^****^ adjusted *p* < 0.00005.

In this section, we aimed to show that GatorST could outperform benchmark methods in spatial domain identification. As illustrated in Figure 2, GatorST consistently outperforms existing benchmark methods in the five evaluation metrics: adjusted Rand index (ARI), normalized mutual information (NMI), clustering accuracy (ACC), purity, and homogeneity. For each dataset, we run every method 10 times with different random seeds and report the mean performance with error bars that capture the variability (Figure 2A). The error bars are modest in magnitude and relatively consistent across datasets, indicating that the performance gains of GatorST are stable. To compare methods across datasets, for each method and dataset we compute the mean score over the 10 runs and summarize these per‐dataset means using boxplots (Figure 2B), which therefore reflect the distribution of performance across datasets. For statistical comparison, we used the mean score across 10 runs as the dataset‐level summary statistic and performed pairwise two‐sided Wilcoxon signed‐rank tests comparing GatorST with each baseline across the 16 benchmark datasets for each clustering metric. Across all five evaluation metrics, GatorST achieves the highest median score with comparable or lower variability than the baselines, demonstrating consistently superior performance. We further add statistical significance annotations to highlight where GatorST outperforms competing methods (see Figure 2 legend for the significance notation). The significance markers show that GatorST significantly outperforms most competing methods on the majority of metrics. These results suggest the effectiveness and robustness of the GatorST in identifying spatially and biologically coherent domains. Specifically, GatorST shows strong performance across all slices of the human dorsolateral prefrontal cortex (DLPFC) dataset (slices #151507 to #151676), which are characterized by complex and heterogeneous tissue architecture. On average, GatorST surpasses all competing state‐of‐the‐art methods by a considerable margin. In particular, GatorST achieves an average improvement in ARI of more than 10% compared to the other contrastive learning‐based methods, GraphST and CCST. This result suggests that GatorST more effectively captures spatial expression patterns, including spatial proximity and gene expression similarity, while preserving the underlying anatomical structure of the tissue. Importantly, the superiority of GatorST is not limited to human brain tissue. Significant performance improvements were observed in the human breast cancer and mouse brain anterior datasets. Across these diverse spatial contexts, GatorST consistently delivers superior performance across all evaluation metrics, demonstrating its generalizability and adaptability to varying spatial resolutions, gene expression profiles, and biological variability.

### 2.3 GatorST Demonstrates Superior Imputation Performance Across Diverse Datasets

**FIGURE 3:**
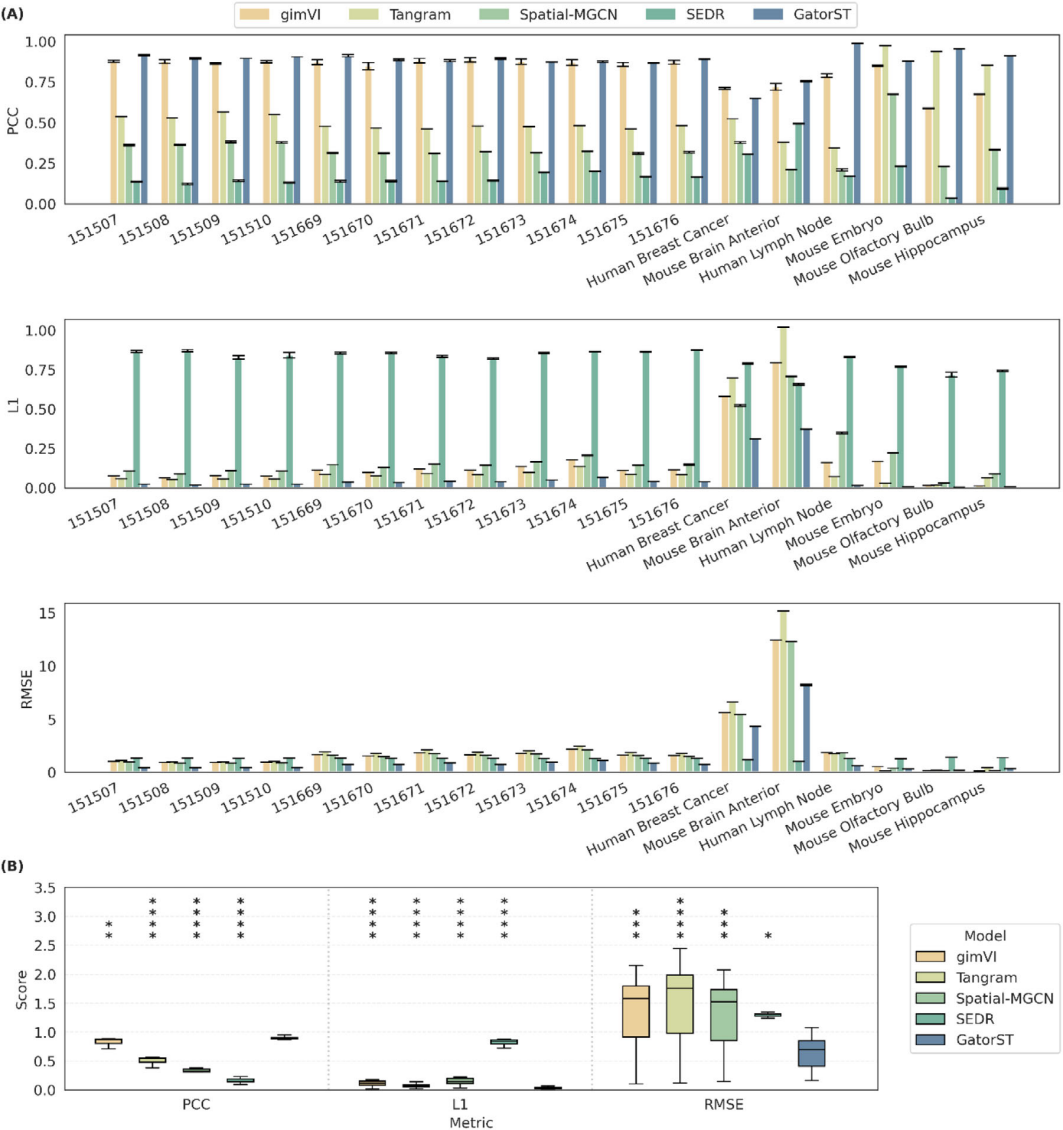
Imputation performance comparison across 18 spatial transcriptomic datasets. (A) For each dataset and method, PCC, L1 loss, and RMSE are shown as mean ± s.d. over 10 runs with different random seeds. (B) Boxplots summarize the distribution of dataset‐level mean scores from panel A across the 18 datasets. Statistical significance was assessed using pairwise two‐sided Wilcoxon signed‐rank tests comparing GatorST with each baseline on the paired dataset‐level mean scores. Higher PCC indicates better imputation performance, whereas lower L1 loss and RMSE indicate better performance. Significance is denoted as n.s., adjusted *p* ≥ 0.05; ^*^ adjusted *p* < 0.05; ^**^ adjusted *p* < 0.005; ^***^ adjusted *p* < 0.0005; ^****^ adjusted *p* < 0.00005.

For gene expression imputation, we compared GatorST with a number of state‐of‐the‐art spatial transcriptomics imputation methods, including SEDR [23], Spatial‐MGCN, gimVI [24], and Tangram [25]. These methods can be classified into two groups: reference‐based methods (i.e., gimVI and Tangram) and reference‐free methods (i.e., SEDR and Spatial‐MGCN). Specifically, gimVI and Tangram are designed to integrate spatial transcriptomics with single‐cell RNA sequencing (scRNA‐seq) data to accurately predict missing gene expression profiles. To ensure a fair comparison among all methods, we utilized their publicly available reference‐free implementations, as these methods typically require matched scRNA‐seq reference data, which may not always be accessible. In contrast, SEDR and Spatial‐MGCN do not require matched scRNA‐seq reference data. As shown in Figure 3A, for each dataset, we run every method multiple times with different random seeds and report the mean performance with error bars that capture the variability. To compare methods across datasets, we then compute, for each method and dataset, the mean PCC, L1, and RMSE over runs and summarize these per‐dataset estimates using boxplots (Figure 3B), which therefore display the distribution of performance across datasets. For statistical comparison, we used the mean PCC, L1 loss, and RMSE across 10 runs as dataset‐level summary statistics and performed pairwise two‐sided Wilcoxon signed‐rank tests comparing GatorST with each baseline across the 18 benchmark datasets for each imputation metric. The error bars are again modest and relatively uniform across datasets, indicating that the imputation performance of GatorST is stable. We additionally annotate statistical significance to highlight where GatorST outperforms competing methods (see Figure 3 legend for the significance notation). GatorST consistently achieved the highest PCC and the lowest L1 and RMSE across the majority of samples, demonstrating strong performance across diverse ST datasets. Notably, SEDR outperformed GatorST in terms of RMSE for the human breast cancer and mouse brain anterior datasets, suggesting that SEDR may have strengths specific to certain datasets. Overall, these results highlight the effectiveness and robustness of GatorST in spatial gene expression imputation tasks, particularly in reference‐free contexts.

### 2.4 GatorST Accurately Reveals Anatomic Layers in Human Prefrontal Cortex

**FIGURE 4:**
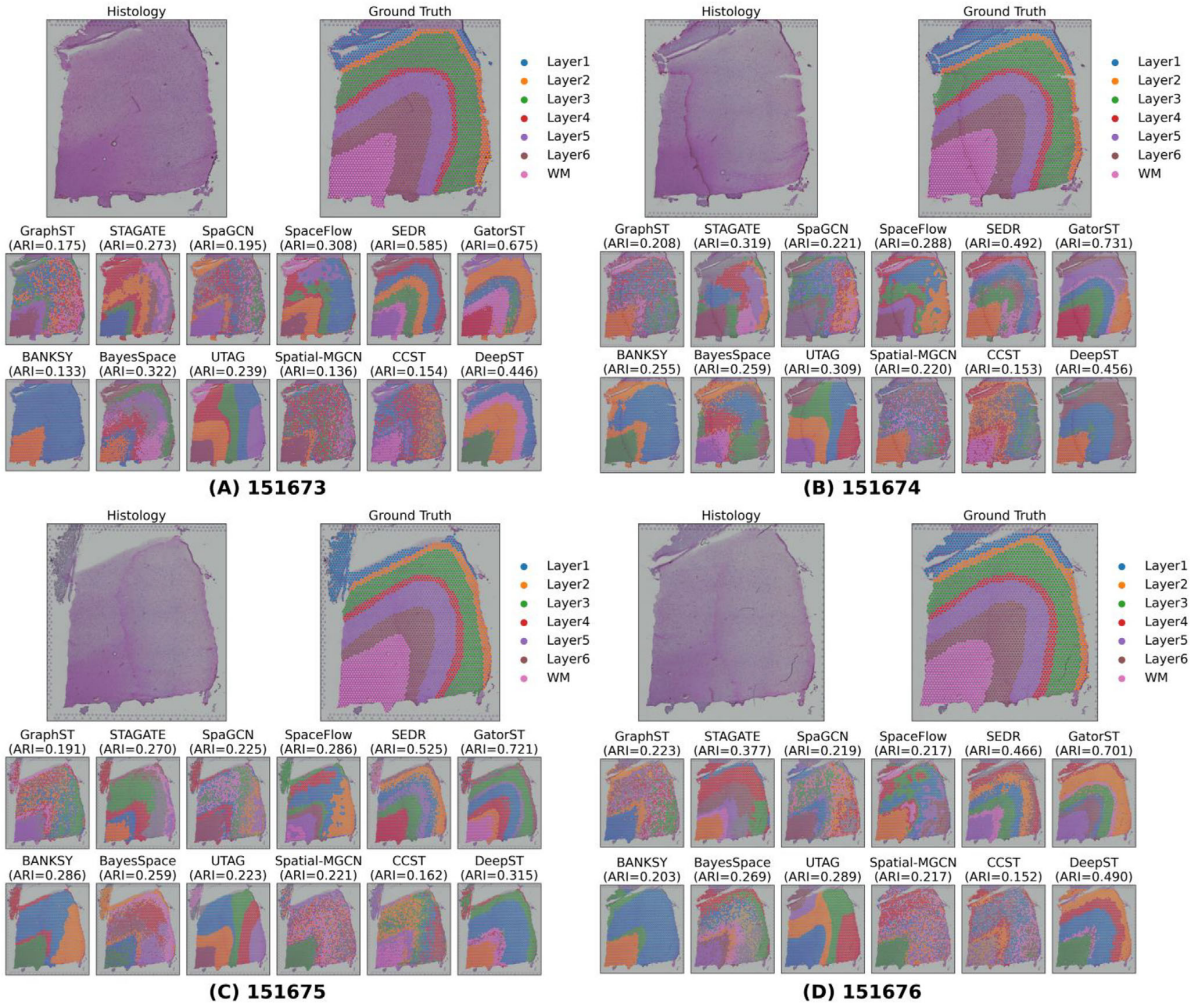
Comparison of manual annotations and clustering outputs for the representative slices #151673 to #151676 from the human DLPFC dataset. All embedding‐based methods are clustered using K‐means for ARI computation; BayesSpace and BANKSY use their built‐in domain inference.

The human DLPFC dataset offers high‐resolution spatially resolved transcriptomic profiles from 12 tissue slices, each capturing the structure of the human DLPFC. These slices include either four or six well‐defined cortical layers, along with the underlying white matter (WM). The availability of detailed annotations makes this dataset a robust benchmark for evaluating the accuracy of spatial domain identification methods in recovering biologically meaningful cortical architecture. To assess the performance of the proposed GatorST and baseline methods, we applied all methods to four consecutive tissue slices from the dataset (slices #151673 to #151676). As shown in Figure 4, GatorST consistently achieved the highest ARI scores across all four slices, with values of 0.661, 0.662, 0.681, and 0.688, respectively. This consistent superiority demonstrates GatorST's ability to effectively delineate cortical layer boundaries across varied tissue architectures. Among the competing methods, SEDR also showed competitive performance, achieving ARI scores of 0.585 on slice #151673 and 0.525 on slice #151675. However, its accuracy varied across slices, suggesting a greater sensitivity to spatial noise or sample‐specific variability. Collectively, these results highlight the superiority of GatorST in spatial domain identification, both in terms of overall accuracy and its adaptability to the structures of cortical tissue.

Beyond these quantitative metrics, we further evaluated how well the inferred spatial domains reflect the known cytoarchitecture of the human prefrontal cortex. As shown in Figure 4, the domains predicted by GatorST form continuous, ribbon‐like bands that closely align with the manually annotated cortical layers, showing sharp transitions at layer boundaries and clear separation between superficial and deep cortical regions. Layer 1 and the white‐matter compartment are accurately localized at the pial and white‐matter margins, respectively, and the intermediate layers display minimal mixing. In contrast, several competing methods produce more irregular or fragmented domain patterns, with partial merging of adjacent layers, blurred inter‐laminar boundaries, and occasional misassignment of white‐matter spots into gray‐matter regions. These findings suggest that GatorST not only achieves superior clustering performance on the DLPFC dataset but also provides a clearer and more anatomically faithful delineation of cortical lamination than existing approaches.

### 2.5 Trajectory Inference Using Spatial Representations

**FIGURE 5:**
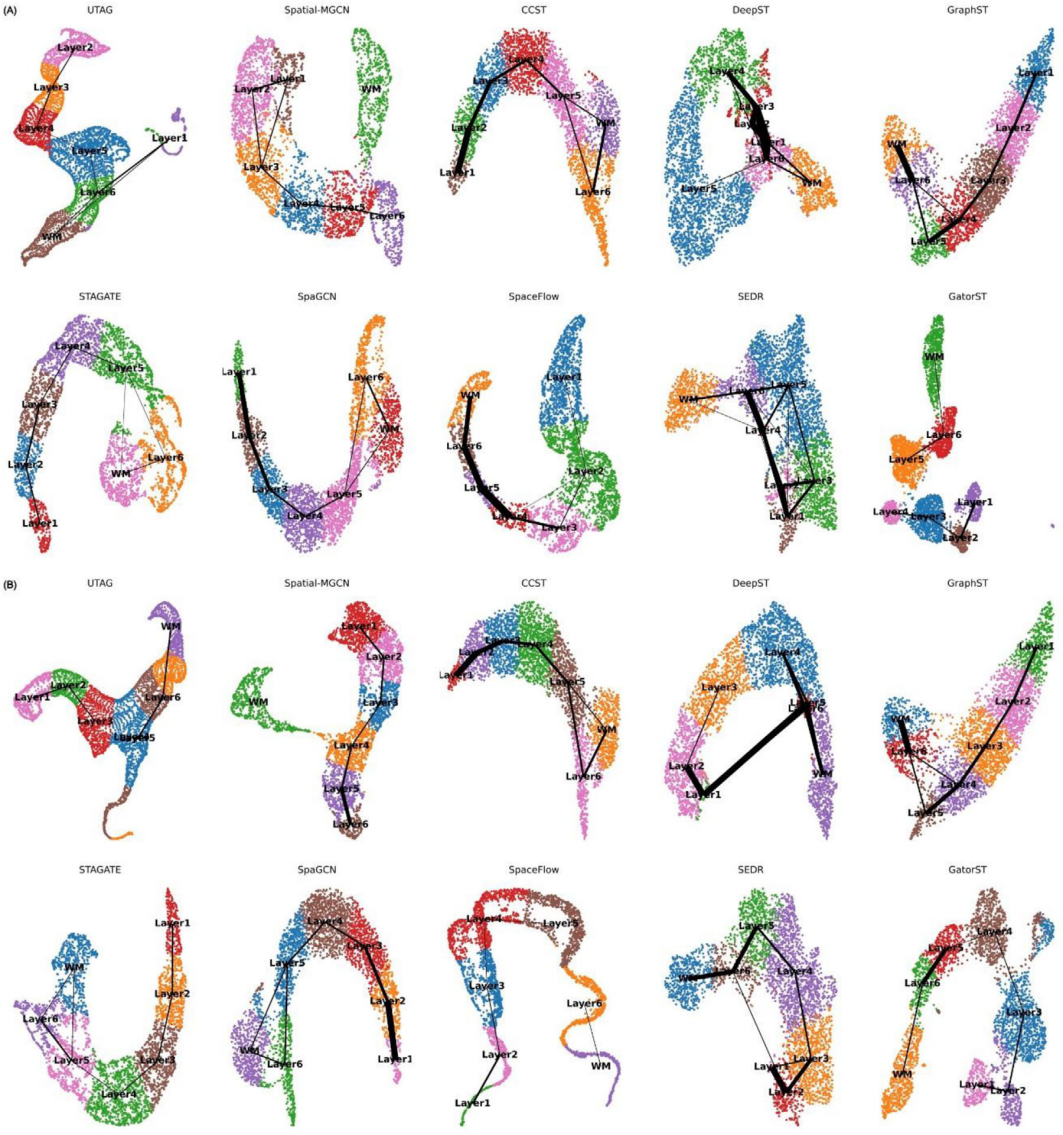
The UMAP and PAGA results of GatorST and baseline methods on human DLPFC slices #151675 and #151676.

To comprehensively evaluate the quality of the learned representations, we applied the proposed GatorST framework to two representative slices (#151675 and #151676) of the human DLPFC dataset. We visualized the learned embeddings using Uniform Manifold Approximation and Projection (UMAP) [26] and compared the outputs with those generated by several state‐of‐the‐art baseline methods. As illustrated in Figure 5, GatorST produces a well‐organized, sequential development of the cortical layers in slices #151675 and #151676 (Panels A and B). In the UMAP visualizations, GatorST displays distinct boundaries and minimal intermixing between adjacent cortical layers, highlighting its ability to preserve anatomical structure in the latent space. Each cortical layer forms a spatially coherent and distinct cluster, with Layer 1 and WM accurately localized at the respective cortical margins. Compared to baseline methods, GatorST reveals a more coherent laminar hierarchy characterized by sharper boundaries, less overlap, and improved separation between layers.

To further evaluate the topological consistency of the latent space, we employed the partition‐based graph abstraction (PAGA) [27], which constructs a graph‐based representation of cluster connectivity based on transcriptomic similarity. In a PAGA graph, nodes represent clusters that correspond to cortical layers, while edges indicate the confidence of transcriptional transitions between clusters. Edge weights quantify the degree of transcriptomic continuity, with stronger connections reflecting gradual biological transitions and weaker or absent connections representing transcriptional boundaries. The PAGA graphs derived from GatorST embeddings reveal a linear, biologically plausible trajectory that reflects the expected spatial progression across cortical layers. This coherent structure contrasts with the PAGA graphs generated by baseline methods, which display fragmented topologies, spurious inter‐cluster connections, or a lack of directional continuity. The well‐defined and interpretable trajectories captured by GatorST further validate its ability to learn biologically meaningful gene expression patterns, reflecting the underlying cytoarchitecture of the human DLPFC.

### 2.6 GatorST Effectively Corrects for Batch Effects

**FIGURE 6:**
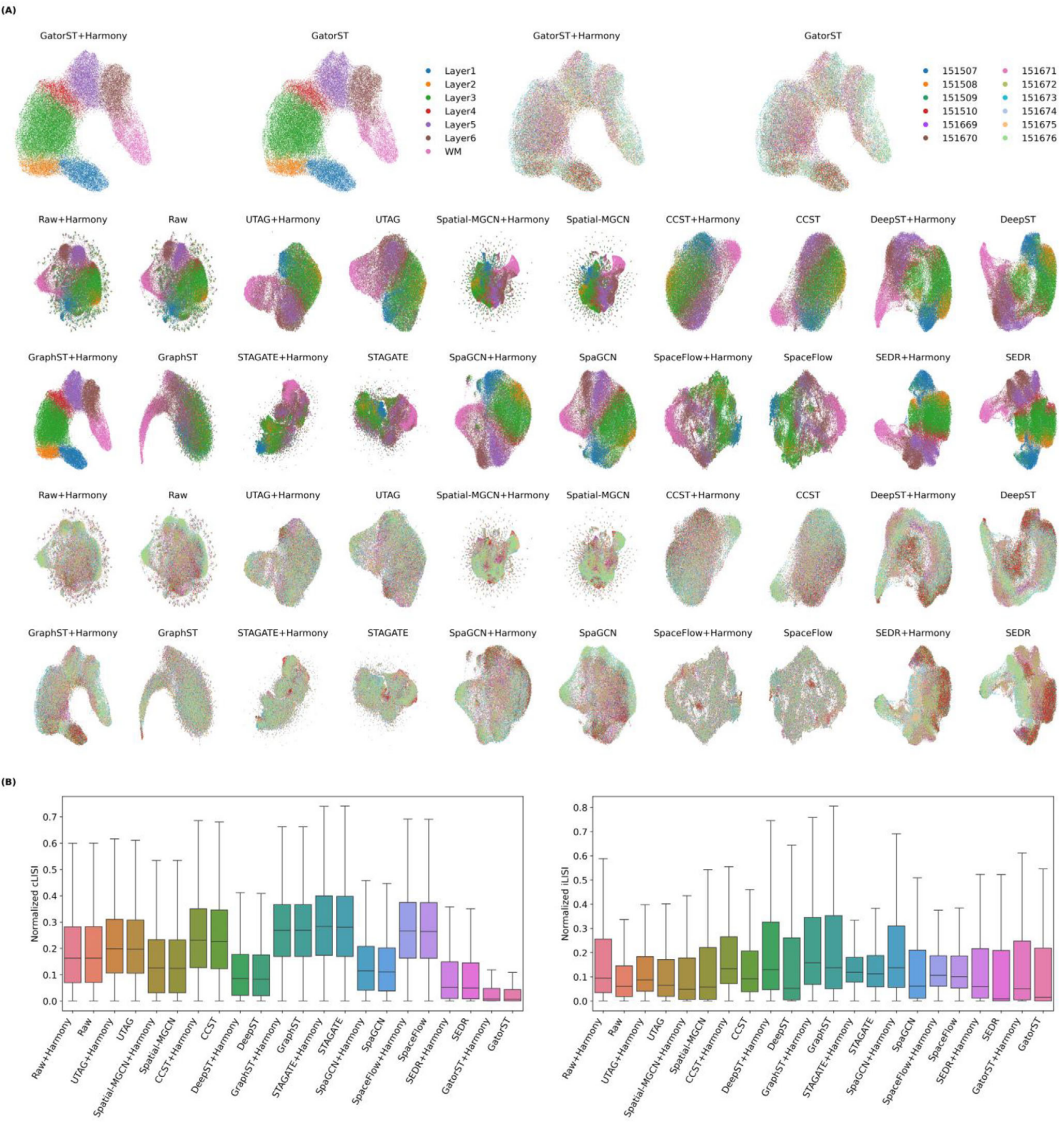
(A) Batch integration results for the human DLPFC dataset across nine baselines and the proposed GatorST method, each shown both with and without Harmony integration. (B) Quantitative assessment of integration performance was conducted using iLISI and cLISI scores. Higher iLISI values indicate better batch integration, while lower cLISI values reflect superior preservation of biological structure.

To address the challenge of batch effects in spatial transcriptomics, we evaluated the use of joint embeddings across multiple batches by projecting them into a shared latent space. We benchmarked ten state‐of‐the‐art methods, including our GatorST, using the human DLPFC dataset to evaluate their effectiveness in preserving biological structure while integrating batch‐specific variations, as illustrated in Figure 6. We utilized Harmony [28] as the batch integration algorithm because of its strong performance in single‐cell data integration [29]. To quantify the performance of batch effect correction, we employed two metrics: cell type LISI (cLISI), which assesses the preservation of biological distinctions such as cortical layers, and the integration LISI (iLISI), which measures the degree of batch integration [28]. Higher iLISI values indicate better integration across batches, while lower cLISI values reflect superior preservation of biological structure.

Specifically, we examined the Raw and Raw+Harmony to assess the impact of Harmony integration on Principal Component Analysis (PCA)‐based embeddings. “Raw” refers to direct dimensionality reduction via PCA without any batch correction, while “Raw+Harmony” applies Harmony to the PCA‐reduced representations. In Figure 6B, there was minimal difference in cLISI scores between the Raw and Raw+Harmony. However, Raw+Harmony demonstrated improved iLISI scores, suggesting enhanced batch integration compared to the uncorrected Raw. Among the baseline methods, STAGATE+Harmony achieved relatively high iLISI scores, indicating effective batch integration. Nonetheless, this improvement came at the expense of blurring biological structures, resulting in embeddings that failed to separate cortical layers and led to spatial ambiguity, as shown in Figure 6A. In contrast, SEDR+Harmony and GatorST+Harmony yielded well‐separated and biologically coherent representations of cortical layers, effectively preserving not only layer‐specific identities but also their developmental progression. While GatorST+Harmony achieved the lowest cLISI across all methods, indicating superior preservation of biologically coherent spatial architecture, its iLISI score was comparatively lower, reflecting a more conservative approach to batch integration. This outcome may be attributed to GatorST's focus on local neighborhood structure and contrastive alignment based on pseudo‐labels, which enhance intra‐class cohesion but may resist excessive integration across batches. Overall, GatorST offers a structure‐preserving approach that prioritizes biological fidelity over aggressive batch integration.

We also systematically evaluated the native (i.e., non‐Harmony) runs for all baseline methods and for GatorST. Except Spatial‐MGCN, incorporation of Harmony consistently increased iLISI, indicating enhanced cross‐batch mixing, whereas its impact on cLISI was method dependent. For GatorST, the native configuration already produced sharply delineated cortical layers, with cLISI values comparable to those of GatorST+Harmony, demonstrating that preservation of biological structure does not rely on external batch correction. Nevertheless, its iLISI remained lower than in the Harmony‐integrated setting, reflecting a more conservative degree of cross‐batch mixing. Collectively, these results indicate that, while Harmony can further improve the extent of batch integration for GatorST, its downstream structural performance (cLISI) is essentially unchanged, suggesting that batch effects are largely accommodated by GatorST's intrinsic modeling capacity.

### 2.7 GatorST Generalizes Across Spatial Transcriptomics Platforms and Tissue Types

To examine whether the performance gains of GatorST extend beyond 10x Visium data, we expanded the benchmark panel to include both Visium and non‐Visium datasets. In addition to the Visium human breast cancer, human lymph node, and mouse brain anterior, we now evaluate GatorST on three non‐Visium datasets: an E9.5 mouse embryo dataset, an adult mouse olfactory bulb dataset, and a mouse hippocampus dataset.

**FIGURE 7:**
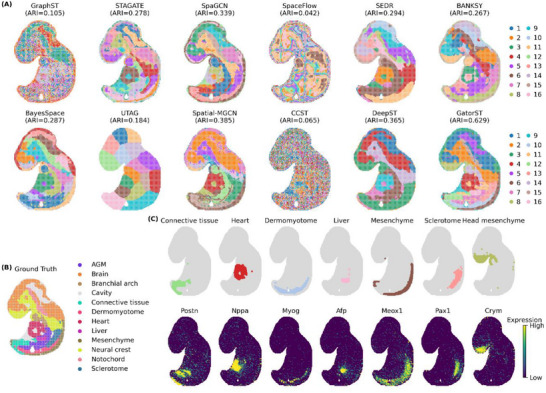
Spatial domain identification in an E9.5 mouse embryo profiled with Stereo‐seq. (A) Clustering results of 11 baseline methods and GatorST, all run with 16 clusters to obtain higher‐resolution tissue segmentation. The ARI of each method is shown in parentheses. (B) Reference anatomical annotations comprising 12 regions: AGM, brain, branchial arch, cavity, connective tissue, dermomyotome, heart, liver, mesenchyme, neural crest, notochord, and sclerotome. (C) Validation using canonical marker genes, with anatomical masks (top) and expression maps (bottom) for Postn (connective tissue), Nppa (developing heart), Myog (dermomyotome), Afp (liver), Meox1 (mesenchyme), Pax1 (sclerotome), and Crym (head mesenchyme).

We first assessed spatial domain identification on an E9.5 mouse embryo profiled with Stereo‐seq. The anatomical annotation defines 12 reference regions: AGM, brain, branchial arch, cavity, connective tissue, dermomyotome, heart, liver, mesenchyme, neural crest, notochord, and sclerotome—yet we set the number of clusters to 16 to obtain a higher‐resolution segmentation of the tissue. Figure 7 compares the outputs of 11 baseline methods and GatorST (panel A), alongside the reference annotation (panel B) and canonical marker maps (panel C; Postn for connective tissue, Nppa for developing heart, Myog for dermomyotome, Afp for liver, Meox1 for mesenchyme, Pax1 for sclerotome, and Crym for head mesenchyme). GatorST attains the highest ARI (0.629), substantially outperforming the next‐best Spatial‐MGCN (0.385) and DeepST (0.365). Consistent with the marker distributions, GatorST yields compact and contiguous domains with smoother boundaries, sharper delineation of cardiac compartments that follow Nppa, and accurate localization of Afp‐positive liver and Postn‐enriched connective tissue, resulting in closer morphological concordance with the anatomical ground truth across major territories.

**FIGURE 8:**
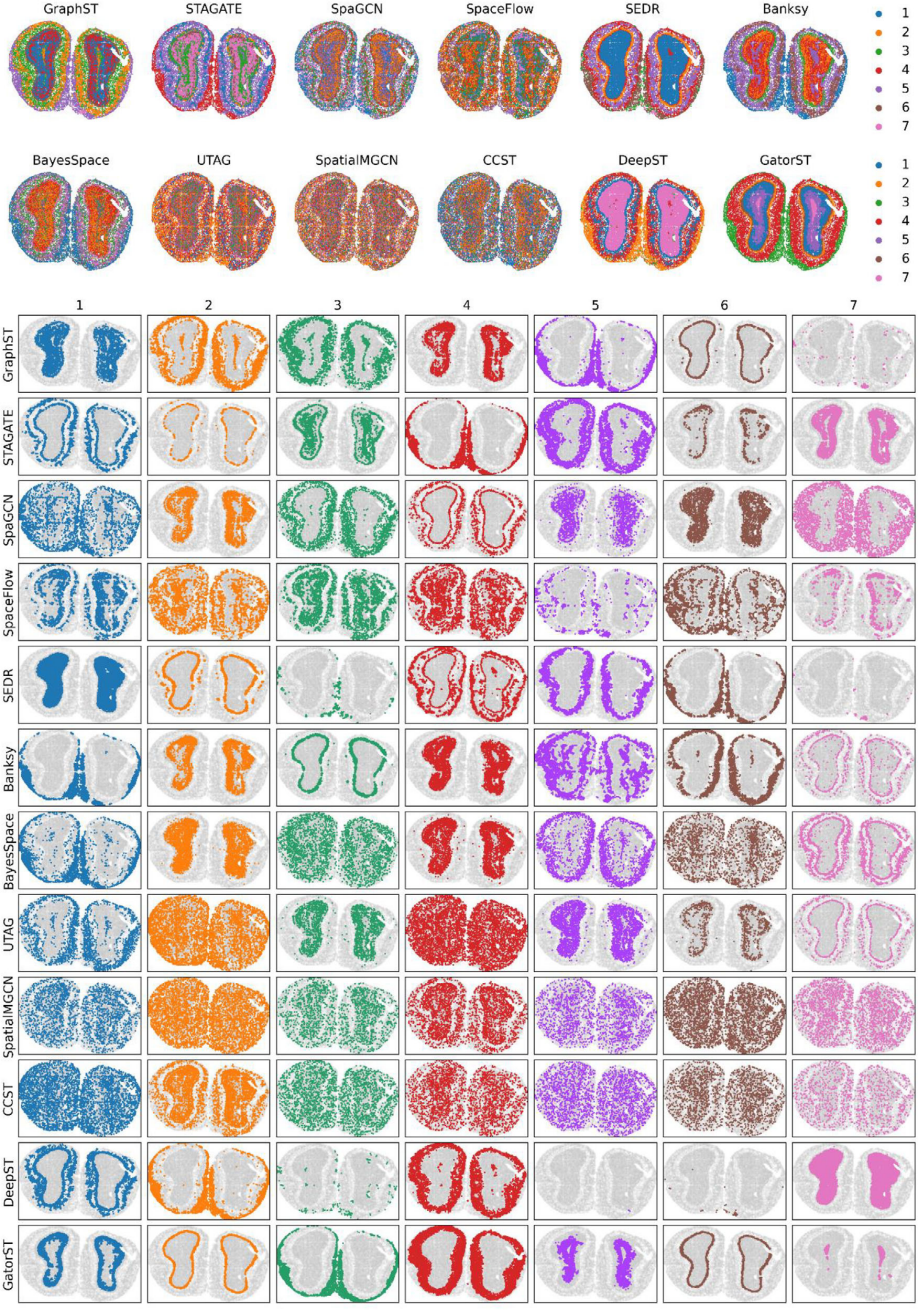
Spatial domain identification in the mouse olfactory bulb profiled with Stereo‐seq. Top row: clustering outputs of 11 baseline methods and GatorST, colored by the seven canonical laminae. Remaining panels: layer‐wise masks for each method overlaid on the histology, showing the internal plexiform layer (IPL, 1), external plexiform layer (EPL, 2), olfactory nerve layer (ONL, 3), glomerular layer (GL, 4), granule cell layer (GCL, 5), mitral cell layer (MCL, 6), and rostral migratory stream (RMS, 7).

We further evaluated GatorST on a non‐Visium Stereo‐seq section of adult mouse olfactory bulb, as shown in Figure 8. Here, GatorST recovers the canonical laminar architecture with sharp, spatially coherent boundaries: IPL (blue, 1), EPL (orange, 2), ONL (green, 3), GL (red, 4), GCL (purple, 5), MCL (brown, 6), and RMS (pink, 7). The assignments align with established OB anatomy in the Stereo‐seq benchmark and with reported marker distributions, indicating biologically meaningful layer delineation. The result demonstrates that our method generalizes across platforms by accurately mapping laminae in a high‐resolution dataset distinct from Visium.

### 2.8 GatorST Resolves Intratumoral Heterogeneity in Human Breast Cancer

**FIGURE 9:**
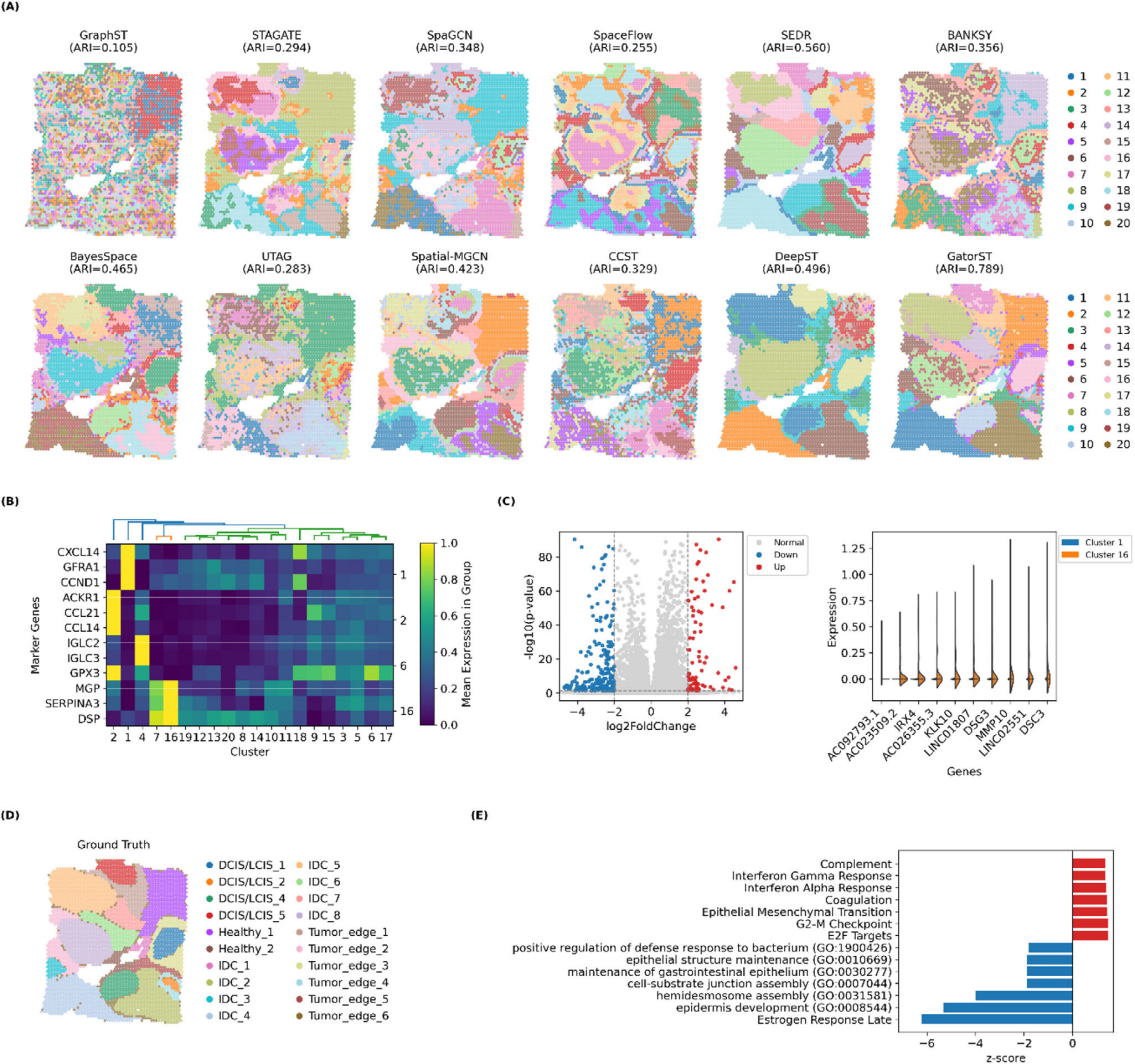
GatorST resolves intratumoral heterogeneity in the human breast cancer dataset. (A) Clustering results of 11 baseline methods and GatorST. (B) Heatmap of the top three differentially expressed genes (DEGs) for each of four GatorST‐defined clusters (Cluster 2: Healthy epithelium; Cluster 16: DCIS/LCIS; Cluster 1: IDC; Cluster 6: Tumor edge. (C) Left, volcano plot of DEGs between the IDC cluster (Cluster 1) and the DCIS/LCIS cluster (Cluster 16), using |log2FoldChange| ≥ 2 and −log10(*p*‐value) < 0.05 as thresholds. Right, violin plots of the top 10 DEGs, highlighting strong enrichment of KLK10 and MMP10 in the DCIS/LCIS cluster. (D) Manual H&E‐based annotation, with 20 regions aggregated into four major compartments: DCIS/LCIS, IDC, healthy epithelium, and low‐malignant tumor edge. (E) Pathway enrichment analysis based on DEGs between IDC and DCIS/LCIS. Positive ***z***‐scores (red bars) indicate pathways upregulated in IDC (e.g., E2F targets, G2/M checkpoint, EMT, interferon response, complement, and coagulation), whereas negative ***z***‐scores (blue bars) indicate pathways upregulated in DCIS/LCIS (e.g., estrogen response and epithelial adhesion/differentiation processes).

To evaluate whether GatorST produces biologically informative embeddings for downstream analysis, we applied it to a human breast cancer Visium dataset and compared its performance with a series of state‐of‐the‐art spatial clustering methods, including GraphST, STAGATE, SpaGCN, SpaceFlow, SEDR, BANKSY, BayesSpace, UTAG, Spatial‐MGCN, CCST, and DeepST. GatorST delineated spatial domains that were more spatially coherent and more concordant with the predefined tissue regions than all competing methods (Figure 9). Consistently, it achieved the highest ARI (0.789), indicating improved agreement with ground‐truth annotations (Figure 9A). Manual hematoxylin and eosin (H&E)‐based annotation followed the scheme of a previous study [23], in which the tissue was subdivided into 20 regions that were further aggregated into four major morphological compartments: ductal carcinoma in situ/lobular carcinoma in situ (DCIS/LCIS), invasive ductal carcinoma (IDC), healthy epithelium, and low‐malignant tumor edge (Figure 9D). In our analysis, these four categories were used as reference compartments. GatorST recovered clusters that closely matched them: Cluster 2 was enriched in healthy epithelium, Cluster 16 corresponded to DCIS/LCIS, Cluster 1 represented IDC, and Cluster 6 localized to tumor‐edge regions.

To further dissect cancer tissue heterogeneity, we examined the expression of the top three differentially expressed genes (DEGs) for each of these four clusters across all spatial domains. The resulting heatmap revealed clearly distinct expression patterns among the Healthy (Cluster 2), DCIS/LCIS (Cluster 16), IDC (Cluster 1), and Tumor‐edge (Cluster 6) compartments (Figure 9B), highlighting substantial molecular heterogeneity within the tumor and its surrounding microenvironment.

We then focused on the transition from in situ to invasive disease by performing a differential expression analysis between the IDC cluster (Cluster 1) and the DCIS/LCIS cluster (Cluster 16). Using |log2FoldChange| ≥ 2 and −log10(*p*‐value) < 0.05 as thresholds, we identified 327 significant DEGs between these two clusters (Figure 9C, left). Among the top DEGs, the kallikrein‐related peptidase KLK10 and the matrix metalloproteinase MMP10 showed markedly higher expression in Cluster 16 than in Cluster 1, as illustrated by violin plots of the top 10 genes (Figure 9C, right). Both genes have previously been implicated in breast cancer: KLK10 is frequently downregulated by CpG island hypermethylation in invasive breast cancers, yet high KLK10 expression in certain subtypes is associated with poor prognosis and tamoxifen resistance [30, 31], whereas MMP10 overexpression has been linked to increased invasion, metastasis, and unfavorable outcomes in multiple tumor types [32, 33]. Their strong enrichment in the DCIS/LCIS‐like cluster suggests that they may serve as candidate markers of pre‐invasive or high‐risk in situ lesions and may contribute to progression toward invasive carcinoma.

To further delineate functional differences between DCIS/LCIS and IDC, we performed pathway enrichment analysis based on DEGs between Cluster 1 and Cluster 16 (Figure 9E). Positive directional *z*‐scores indicate pathways upregulated in IDC, whereas negative scores correspond to pathways upregulated in DCIS/LCIS. IDC (Cluster 1) showed significant enrichment of Hallmark E2F targets and G2/M checkpoint gene sets, as well as epithelial–mesenchymal transition (EMT), interferon‐α/γ response, complement and coagulation pathways, pointing to increased cell‐cycle activity, enhanced migratory and invasive potential, and a more inflammatory or immune‐activated tumor microenvironment. In contrast, DCIS/LCIS (Cluster 16) was enriched for estrogen response and several epithelial adhesion– and differentiation‐related processes, including epidermis development, hemidesmosome assembly, cell–substrate junction assembly, and epithelial structure maintenance, consistent with an in situ lesion that largely preserves epithelial architecture and exhibits lower invasive capacity.

Collectively, these results demonstrate that GatorST resolves spatial domains that are highly concordant with pathologist‐defined regions in human breast cancer but also reveals nuanced molecular distinctions among healthy epithelium, DCIS/LCIS, IDC, and tumor‐edge compartments. By integrating spatial organization, gene expression profiles, and pathway‐level perturbations, GatorST enables a refined dissection of cancer tissue heterogeneity and provides mechanistic insights into the progression from in situ to invasive breast carcinoma.

### 2.9 Parameter Sensitivity Analysis of the Proposed GatorST Framework

**FIGURE 10:**
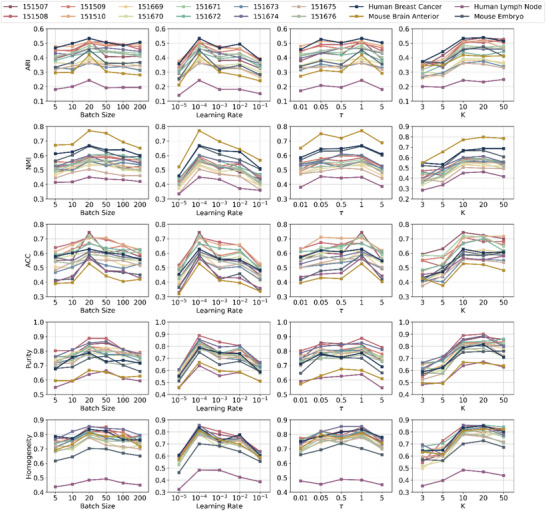
Hyperparameter analysis results using five clustering evaluation metrics: ARI, NMI, ACC, purity, and Homogeneity on 16 spatial transcriptomic datasets. The learning rate, the batch size, and the temperature parameter τ used in the contrastive learning objective, as well as the number of clusters K, were carried out in the analysis. Higher values are better for all these metrics.

To further assess the effectiveness of GatorST under varying training conditions, we conducted a comprehensive parameter sensitivity analysis focusing on three critical hyperparameters: the learning rate, the batch size, and the temperature parameter τ used in the contrastive learning objective, and the number of clusters K. In particular, the learning rate plays a key role in the convergence speed and overall optimization. We evaluated clustering performance across a broad range of learning rates from 10^−5^ to 10^−1^. As shown in Figure 10, performance was highly sensitive to this parameter. In particular, clustering performance improved with increasing learning rates up to a threshold, after which further increases led to performance degradation. Next, we varied the batch size from 5 to 200 to examine its impact on clustering performance. The results indicated that intermediate batch sizes, particularly in the range of 20 to 50, consistently yielded higher ARI and NMI scores, providing an effective balance between clustering performance and training stability. The temperature parameter τ is a critical parameter in the contrastive learning objective, as it controls the sharpness of the similarity distribution and influences the separation of positive and negative sample pairs within the embedding space. We tested various values of τ, including 0.01, 0.05, 0.5, 1, and 5. Looking at Figure 10, moderate values of τ (e.g., 0.5 ∼ 1) achieved optimal performance across all evaluation metrics. Lower values, such as τ = 0.01 excessively amplified contrastive penalties, leading to suboptimal performance, while higher values, such as τ = 5 overly smoothed the similarity distribution, reducing the GatorST's ability to discriminate between representations. Finally, we investigated the sensitivity to K by varying K ∈ {3, 5, 10, 20, and 50}. Using too small a K (e.g., K ≤ 5) degraded clustering performance, likely because the pseudo‐labels under‐segment the underlying structure. In contrast, performance was relatively stable once K ≥ 10, and increasing K beyond 10 did not bring consistent improvement. Based on these observations, we fix K = 10 for all datasets to obtain strong performance while avoiding heavy hyperparameter tuning. Overall, the results of the parameter sensitivity analysis indicate that careful selection of the learning rate, batch size, temperature parameter, and the number of clusters K is critically important for improving the clustering performance of GatorST. Most importantly, GatorST maintains adaptability across a broad range of hyperparameter settings, highlighting its practical utility in ST data analysis.

### 2.10 Ablation Studies of the Proposed GatorST Framework

To evaluate the contributions of the core components within the GatorST framework, we conducted extensive ablation studies across all benchmark datasets and clustering evaluation metrics. Specifically, we studied the following three variants: (i) GatorST_α_, where the contrastive learning objective was omitted; (ii) GatorST_β_, where the subgraph extraction was excluded; (iii) GatorST_γ_, where the (GCN) [34] was replaced with a Graph Attention Network (GAT) [35].

**FIGURE 11:**
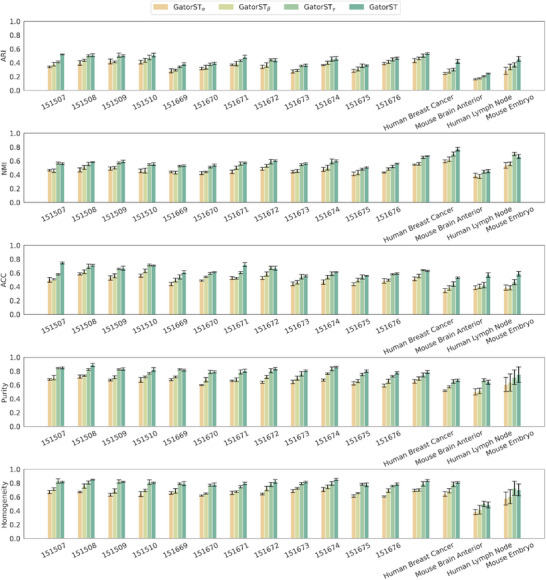
Clustering performance comparison across 16 spatial transcriptomic datasets between GatorST and its three variants.

The results of the ablation study are shown in Figure 11. Among the three variants, GatorST_α_ displayed the most significant and consistent decline in clustering performance across the five metrics used. On average, the ARI decreased by more than 10%, with particularly significant declines observed in DLPFC slices 151507 and 151671, as well as in the mouse brain anterior dataset. Similarly, both NMI and Homogeneity showed approximately a 10% decrease. These results emphasize the crucial role of contrastive learning in aligning intra‐class embeddings and maximizing inter‐class distinction.

GatorST_β_ demonstrated consistent yet moderate performance declines across datasets. While the decline in ARI was less severe compared to GatorST_α_, significant declines were observed in NMI, ACC, Purity, and Homogeneity, particularly in DLPFC slices 151510 and 151671, as well as in the mouse brain anterior dataset. For instance, both NMI and Homogeneity decreased by approximately 10%, highlighting the importance of incorporating localized structural context through two‐hop subgraph extraction. These results suggest that subgraph‐based modeling is vital for capturing microenvironmental relationships and preserving the spatial coherence of learned domains.

GatorST_γ_ yielded mixed outcomes. In certain DLPFC slices, like 151669, minor improvements in Purity and Homogeneity were observed. However, these improvements were inconsistent across datasets, with declines in Purity and Homogeneity observed in both the human breast cancer and mouse brain anterior datasets. ARI and NMI showed minor variations compared to GatorST for most datasets. While the GAT may introduce benefits in specific spatial contexts through its attention mechanisms [36], it does not consistently deliver optimal clustering performance. In contrast, GCN provides a more balanced and efficient approach for modeling spatial transcriptomic data within the GatorST framework.

The results of the ablation study highlight the importance of the core components of GatorST. Both contrastive learning (GatorST_α_) and subgraph‐based modeling (GatorST_β_) are critical for achieving improved clustering performance. These findings support the design rationale of GatorST, which integrates the capture of spatial topology, gene expressions, and the learning of discriminative feature representations into a unified framework for spatial domain identification.

### 2.11 The Trade‐Offs Between Performance and Computational Efficiency

**FIGURE 12:**
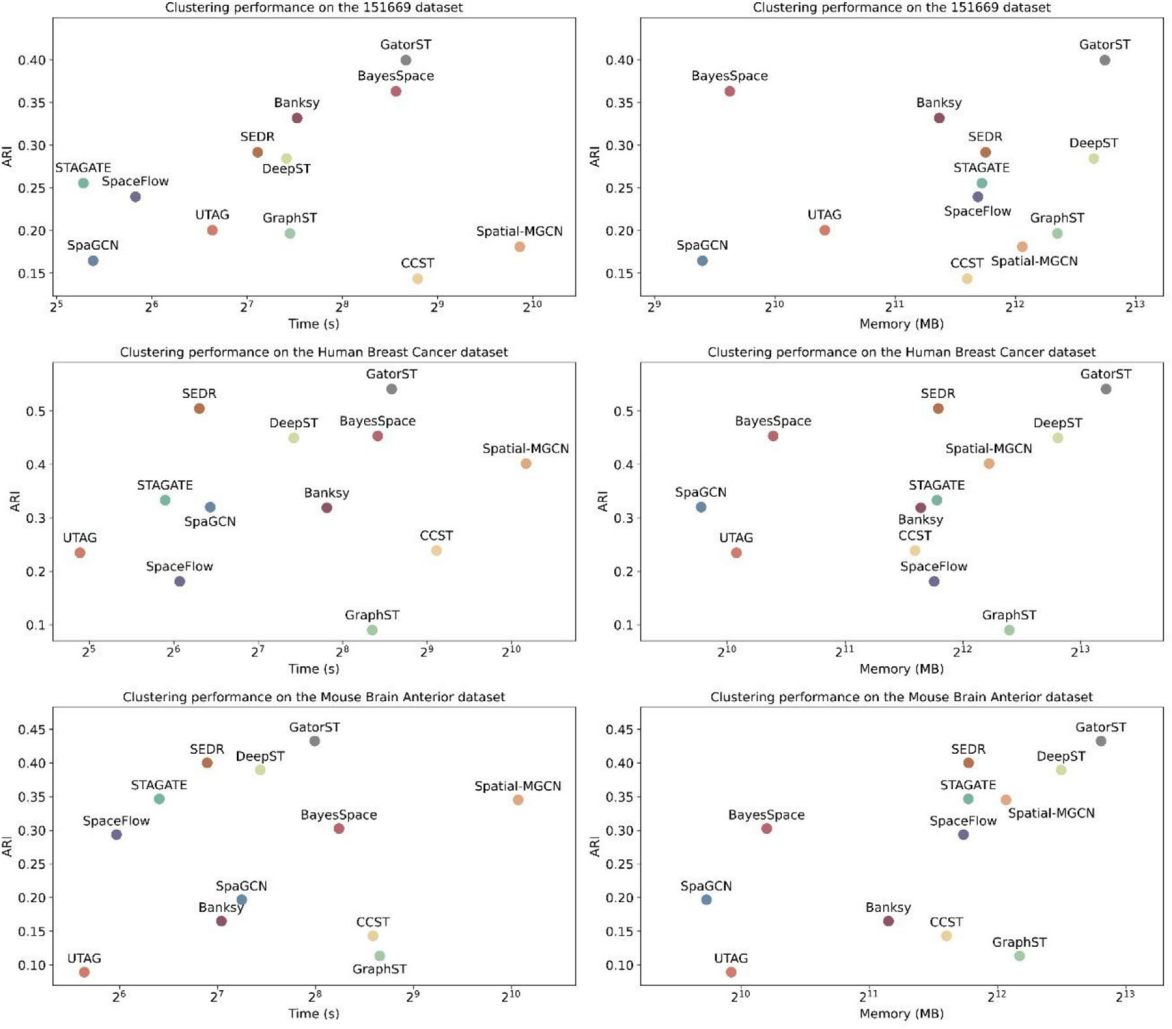
Clustering performance, runtime, and memory usage of baseline methods and our proposed GatorST across the slice #151669 of the human DLPFC dataset, human breast cancer, and mouse brain anterior tissue. Each scatter plot illustrates the trade‐offs between clustering accuracy (measured by ARI) and computational efficiency.

We evaluated the runtime and memory usage of all baseline methods and our GatorST using three datasets: slice #151669 of the human DLPFC dataset, human breast cancer, and mouse brain tissue. As shown in Figure 12, GatorST consistently achieved the highest ARI scores across all datasets, with ARI values of 0.3998 for slice #151669, 0.5415 for human breast cancer, and 0.4327 for mouse brain anterior, demonstrating its superior clustering accuracy. In terms of computational efficiency, GatorST demonstrated competitive runtime performance for all datasets. Notably, its runtime was consistently shorter than that of Spatial‐MGCN and CCST, both of which showed longer runtimes despite delivering lower clustering accuracy. While GatorST showed relatively higher memory usage compared to most baseline methods (typically ranging from 2^12^ to 2^13^ MB), it effectively utilized these additional computational resources to deliver improved clustering results. For instance, in the human breast cancer and mouse brain tissue datasets, GatorST, despite its larger memory footprint, outperformed SpaGCN and UTAG, which had lower resource demands but yielded suboptimal ARI scores. These results highlight a significant trade‐off: although GatorST requires greater memory, it leverages that capacity to achieve state‐of‐the‐art clustering performance while maintaining runtime efficiency. This balance between clustering accuracy and computational cost makes GatorST a powerful tool for studying spatial domain identification. Nevertheless, it is important to acknowledge that GatorST's high memory requirements may limit its applicability in resource‐constrained environments.

## 3 Discussion

In this study, we present GatorST, a versatile and scalable contrastive meta‐learning framework specifically designed for analyzing ST data. GatorST effectively addresses the key challenges in ST data, such as high dimensionality, sparsity, and technical noise, by integrating localized spatial interactions with global gene expression semantics. To capture fine‐grained spatial topology, GatorST constructs two‐hop neighborhood subgraphs, which are enhanced through cluster‐aware pseudo‐labeling obtained via k‐means clustering. The pseudo‐labeling, which serves as weak supervision, improves the contrastive learning process, promoting both spatial coherence and biological relevance in the learned representations. Moreover, GatorST employs an episodic training strategy, enabling adaptive representation learning across various spatial resolutions, gene expression profiles, and tissue architectures.

We validated the effectiveness of GatorST through comprehensive experiments on a broad panel of spatial transcriptomics datasets spanning multiple tissue types, species, and platforms, including human DLPFC, human lymph node, human breast cancer, mouse brain anterior tissue, an E9.5 mouse embryo (Stereo‐seq), adult mouse olfactory bulb (Stereo‐seq), and mouse hippocampus (Slide‐seqV2). Across these benchmarks, GatorST consistently outperforms state‐of‐the‐art methods in key downstream tasks, including spatial domain identification, gene expression imputation, trajectory inference, batch effect correction, and the dissection of intratumoral heterogeneity, highlighting its utility as a general framework for deciphering spatial organization and recovering transcriptomic signals in complex ST data. A detailed parameter sensitivity analysis provides valuable insight into the internal dynamics of GatorST and how different parameter selections impact its performance. The results obtained from the ablation study further demonstrate the efficacy of the GatorST framework. When contrastive learning or subgraph‐based modeling was removed, clustering performance decreased. These findings emphasize the critical roles of both contrastive learning and subgraph‐based modeling in capturing biological and spatial signals within ST data.

With regard to the research methods, this study has several strengths. (i) The proposed GatorST framework effectively integrates local spatial interactions and global gene expression semantics by modeling localized neighborhoods through graph‐based substructures and deriving high‐level biological patterns via k‐means clustering. This combined contextual representation mitigates spatial biases and improves the biological relevance of the clustering outcomes. (ii) Instead of relying on simplistic corruption strategies or heavy graph data augmentation, GatorST introduces pseudo‐label supervision into contrastive learning. This approach strengthens intra‐class cohesion and ensures meaningful separation between classes. (iii) The incorporation of an episodic training strategy inspired by meta‐learning enables GatorST to adapt to variations in spatial resolution, gene expression profiles, and biological heterogeneity. This adaptability improves generalization across diverse ST datasets. (iv) The joint optimization of contrastive and cross‐entropy losses encourages the learning of embeddings that are not only spatially structured but also task‐adaptive. This dual‐objective approach reduces overfitting and improves the robustness of the model in complex biological contexts.

Although GatorST showed strong performance across multiple downstream tasks, these applications serve distinct analytical purposes and should be interpreted accordingly. In GatorST, gene expression imputation is primarily formulated as a predictive task to assess the quality of the learned latent representations, whereas biological interpretation is mainly based on the embeddings themselves and the spatial domains derived from them. Because imputation may introduce smoothing effects, functional differences between raw and imputed expression matrices should be interpreted with caution. A more systematic comparison of enrichment results from raw and imputed data may offer additional insight into the biological impact of imputation and merits further investigation in future studies. The interpretation of trajectory inference depends strongly on the structure of the dataset under study. In this work, PAGA analysis on the DLPFC embeddings was primarily used to evaluate whether GatorST preserves ordered laminar continuity in the latent space, rather than to infer a definitive developmental lineage. By contrast, the E9.5 mouse embryo represents a single developmental stage with multiple concurrent morphogenetic programs, making trajectory inference inherently more difficult to interpret in the absence of temporal resolution. Extending the evaluation of GatorST to temporally resolved developmental datasets, or to embryo datasets with independent lineage annotations, would enable a more rigorous assessment of its utility for trajectory‐related applications.

Although this study has successfully demonstrated the effectiveness of GatorST across multiple benchmarks, its performance on large‐scale ST data needs to be systematically investigated. Recent advances in high‐throughput spatial transcriptomics technologies have led to the generation of large datasets, which pose significant computational and modeling challenges. The key aspects of dealing with such large‐scale data highlight the need for scalable graph construction, memory‐efficient continual learning, and maintaining embedding quality across spatial domains with substantial variability in size, density, and topological complexity. Future studies should incorporate advanced techniques such as graph sparsification, hierarchical representation learning, and scalable mini‐batch contrastive optimization. These adaptations will be crucial for ensuring the effectiveness of GatorST when handling increasingly large and complex ST datasets.

## 4 Methods

### 4.1 Spot‐Spot Graph Structure Learning

Let G=(V,E,X)=(A,X) represent a spot‐spot graph. In the graph, $V=\{v_{1},v_{2},\text{…},v_{C}\}$ represents the set of nodes/spots, where *C* is the total number of spots. E represents the set of edges that connect the nodes. $X\in R^{C\times g}$ is the gene expression matrix obtained after preprocessing, and *g* is the total number of genes. The adjacency matrix $A\in {\left\{0,1\right\}}^{C\times C}$ encodes the connectivity of the graph, where **A**_*ij*_ indicates the presence or absence of an edge between spots *i* and *j*.

To construct the spot‐spot graph G, a similarity score for each pair of nodes is calculated and then used to define the entries in the adjacency matrix. The adjacency matrix **A** can be formalized as follows:

$$\;A_{ij}=\left\{\begin{array}{cc} 1, & \mathrm{if}\;s\left(v_{i},v_{j}\right)\in \mathrm{Top}-k\left({\left\{s\left(v_{i},v_{k}\right)\right\}}_{k=1}^{\left|V\right|}\right),\\ 0, & \mathrm{otherwise}, \end{array}\right.$$

Up to *k* nearest neighbors (i.e., nodes with the highest similarity scores) are selected for each node, and edges are established to connect them. The similarity score *s*(*v_i_*,*v_j_*) between the nodes *v_i_* and *v_j_* is calculated using their spatial locations. The spatial location of a node *v_i_* is represented by a location vector $z_{i}\in R^{z}$, where *z* represents the corresponding dimensionality. Accordingly, the similarity score between two nodes is calculated as follows:

$$s\;\left(v_{i},v_{j}\right)=\left\langle z_{i},z_{j}\right\rangle ,$$

where **z**_*i*_ and **z**_*j*_ are the location vectors of *v_i_* and *v_j_*, respectively.

Based on the foundation established by the spot‐spot graph G, a subgraph is extracted for each node to represent its structural relationships. In particular, the subgraph for a given node consists of its two‐hop neighboring nodes. We also tested incorporating 3‐hop neighboring nodes; however, this did not improve performance and increased computational costs. We take an example of a node *v_i_*, and its subgraph $G_{v}$ can be defined as follows:

$$\;G_{v}=\left(V_{v},E_{v},X_{v}\right),\;\mathrm{where}\;V_{v}=\left\{v\right\}\cup N_{v},$$

where $N_{v}$ is the set of two‐hop neighboring nodes of *v* can be further written as follows:

$$\;N_{v}=\left\{u|1\leq d\left(v,u\right)\leq 2\right\},$$

$$\;E_{v}=\left\{\left(i,j\right)|\;A_{i,j}=1,v_{i},v_{j}\in V_{v}\right\},$$

where the gene expression data for the nodes is represented by the matrix $X_{v}\in R^{N_{v}\times g}$. Here, *d*(*v*, *u*) denotes the distance of the shortest path between nodes *u* and *v*. As such, the process of extracting two‐hop neighbors of node *v* is to select all nodes with a shortest path distance (to node *v*) of less than or equal to 2. Thus, this process can be described as $N_{v}=\left\{u|1\leq d\left(v,u\right)\leq 2\right\}$. Here, $N_{v}=\left|V_{v}\right|$ is the number of nodes, and *g* is the dimensionality of the gene expression features. To learn the representation of the node *v*, a Graph Convolutional Network [34] is applied to its subgraph $G_{v}$ as follows:

$$\;h_{v}=\;\mathrm{GCN}\left(V_{v},E_{v},X_{v}\right),$$

where $h_{v}\in R^{d_{h}}$ is the learned representation of node *v* and *d_h_* the dimensionality of the representation. The learned node representations derived from subgraph‐based graph convolution are subsequently utilized for various downstream tasks, such as spatial domain identification. To further enhance the quality and generalizability of these representations, we introduce a contrastive meta‐learning framework, which integrates both local graph structure and global expression patterns into a unified optimization framework. The details of this framework are described in the following subsection.

### 4.2 Contrastive Meta‐Learning

In this subsection, we present a contrastive meta‐learning framework that leverages cluster‐aware pseudo‐labels as weak supervision to guide the alignment of spot embeddings. This design not only improves intra‐cluster coherence and inter‐cluster separation but also facilitates robust generalization across spatial contexts. In the following subsections, we detail the two key components of this framework: (i) the generation of pseudo‐labels via soft K‐means clustering, and (ii) an episodic training strategy that jointly optimizes contrastive and classification objectives.

### 4.3 Cluster‐Aware Pseudo‐Labeling

Given the graph representation G=(V,E,X)=(A,X), each node v∈V is assigned a pseudo‐label by applying the *K*‐means clustering to the node feature matrix **X**. The goal of *K*‐means clustering is to partition the nodes into *K* distinct clusters by minimizing the sum of squared distances between each node and its assigned cluster centroid.

Let $\left\{\mu_{1},\mu_{2},\text{…},\mu_{K}\right\}$ represent the set of *K* cluster centroids and $\sigma_{k}^{2}$ represent the variance associated with cluster *k*. Each node *v_i_* has an associated probability distribution over the clusters as follows:

$$\;p_{i,k}=\frac{\exp\left(-\frac{1}{2}∥x_{i}-\mu_{k}{∥}^{2}/\sigma_{k}^{2}\right)}{\sum_{j=1}^{K}\exp\left(-\frac{1}{2}∥x_{i}-\mu_{j}{∥}^{2}/\sigma_{j}^{2}\right)}\;,$$

where *p*_*i*,*k*_ represents the probability that node *v_i_* belongs to cluster *k*, ensuring $\sum_{k=1}^{K}p_{i,k}=1$. Instead of applying conventional hard assignments to cluster membership, the cluster centroids are updated based on the soft assignments derived from the probabilities:

$$\;\mu_{k}=\frac{\sum_{i=1}^{\left|V\right|}p_{i,k}x_{i}}{\sum_{i=1}^{\left|V\right|}p_{i,k}}\;.$$

Accordingly, the clustering process consists of two main iterative steps: (i) computing soft assignments. *p*_*i*,*k*_ and (ii) updating the centroid updates μ_*k*_. These steps are repeated until convergence is reached. Finally, each node receives a continuous pseudo‐label in the form of a cluster membership probability vector as follows:

$$\;y_{i}=\left(p_{i,1},p_{i,2},\text{…},p_{i,K}\right)\;,\;\forall v_{i}\in V.$$

We further consider *y_i_* as the hard‐coded label of *v_i_*:

$$\;y_{i}=\;\arg\max_{j}p_{i,j},\;j=1,2,\text{…},K.$$

The resulting *y_i_* serves as the pseudo‐label for each node. Through this pseudo‐label generation process, we establish a weak supervision signal based on soft clustering. These pseudo‐labels are then utilized during meta‐training to guide the joint optimization of node representations through both contrastive and classification objectives.

### 4.4 Multi‐Objective Optimization

With the pseudo‐labels at hand, we are able to build an episodic training strategy that consists of multiple meta‐training tasks. Meta‐learning, often referred to as “learning to learn", trains the model to efficiently adapt to new tasks by optimizing across a distribution of tasks rather than a single task [37, 38, 39]. This approach is particularly well‐suited for ST data analysis, as it enables the model to generalize across diverse spatial and biological contexts, addressing the inherent variability in sample distributions. To be specific, we begin by sampling a support set S and a query set Q to establish a meta‐training task T. The support set S consists of *N* randomly sampled classes from the *K* classes (i.e., clusters), with up to *M* nodes randomly selected from each of these *N* classes. Accordingly, S is also known as a *N*‐way, *M*‐shot support set. In the same vein, the query set Q consists of up to *Q* nodes from the same *N* classes, and these sampled nodes are distinct from those in S. By doing so, each meta‐training task can be established as follows:


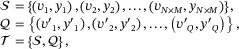

where *v_i_* (or *v*′_*i*_) represents a node in V and *y_i_* (or *y*′_*i*_) is the pseudo‐label associated with *v_i_* (or *v*′_*i*_).

During each episode, a two‐step optimization process is implemented. First, contrastive learning is integrated with pseudo‐labeling within each meta‐training task T. Let $v_{i}^{j}$ represent the *j*‐th node in the *i*‐th class within the support set S, with its learned representation represented as $h_{i}^{j}$, where i=1,2,…,N and j=1,2,…,M. Accordingly, the contrastive loss for the support set can be written as follows:

$$L_{CT}\;\left(S\right)=-\frac{1}{NM}\sum_{i=1}^{N}\sum_{j=1}^{M}\log\frac{\exp\left(h_{i}^{j}\cdot {\hat{h}}_{i}/\tau \right)}{\sum_{k=1,k\neq i}^{N}\exp\left(h_{i}^{j}\cdot {\hat{h}}_{k}/\tau \right)},$$

where ${\hat{h}}_{i}=\frac{1}{M}\;\sum_{j=1}^{M}h_{i}^{j}$ represents the representation of the *i*‐th class, calculated by averaging the representations of *M* nodes that belong to the *i*‐th class. The temperature parameter τ adjusts the sharpness of the similarity distribution within the contrastive objective. Here, we did not use l2‐normalization to preserve more information in the learned representations. By minimizing contrastive loss $L_{CT}$, We encourage the alignment of node embeddings that share the same pseudo‐label while maximizing the separation between embeddings of nodes with different pseudo‐labels.

Second, for the nodes in the query set, task‐specific classification is carried out by assigning each node a label from the *N* available classes. This classification enables the fine‐tuning of the learned representations, ensuring effective adaptation to the specific meta‐training task T. Accordingly, the classification loss can be calculated as follows:


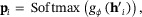

$$L_{CE}\;\left(Q\right)=-\frac{1}{\left|Q\right|}\sum_{i=1}^{\left|Q\right|}\sum_{j=1}^{N}y_{i}^{\left(j\right)}\log p_{i}^{\left(j\right)},$$

where $p_{i}\in R^{N}$ represents the probability distribution over the *N* classes in the meta‐training task T for the *i*‐th query node *v*′_*i*_ in Q. A note of caution is due here $y_{i}^{\left(j\right)}$ is the pseudo‐label value with respect to the *j*‐th class among the *N* classes. The value $p_{i}^{\left(j\right)}$ corresponds to the *j*‐th component of **p**_*i*_.

By combining the contrastive loss $L_{CT}$ on S and the classification loss $L_{CE}$ On Q, the final optimization objective for the meta‐training task T can be formalized as follows:

$$L\;\left(T\right)=\alpha \cdot L_{CT}\left(S\right)+\left(1-\alpha \right)\cdot L_{CE}\left(Q\right),$$

where α∈[0,1] is a hyperparameter that balances the contributions of both losses. Through the training process of contrastive meta‐learning, we obtain node embeddings that are both structurally coherent and semantically discriminative. These representations can be directly applied to various spatial transcriptomics analysis tasks, including spatial domain identification, gene expression imputation, trajectory inference, and batch effect removal. In the following sections, we detail the implementation of each task.

### 4.5 Application Tasks

### 4.6 Spatial Domain Identification

Once the GatorST framework is properly trained, the learned spot representations can be utilized for the task of spatial domain identification. To achieve this, we adopt a clustering‐based strategy that partitions all learned representations into distinct clusters, each corresponding to a spatial domain. Let $H\in R^{C\times d_{h}}$ denote the matrix of learned spot representations, where *C* is the number of spots and *d_h_* is the dimensionality of each representation. Accordingly, we apply K‐means clustering to **H** by optimizing the following objective function:

$$L_{\mathrm{cluster}}=\sum_{i=1}^{C}h_{i}-{\mu_{z_{i}}}_{2}^{2}$$

where $h_{i}\in R^{d_{h}}$ is the representation of the *i*‐th spot (i.e., the *i*‐th row of **H**), $z_{i}\in \left\{1,\text{…},K\right\}$ is the cluster assignment of spot *i*, and $\mu_{z_{i}}$ is the centroid of the *z_i_*‐th cluster. This objective function is used to encourage each representation to be close to the centroid of its assigned group, effectively grouping spots with similar features into the same spatial domain.

### 4.7 Gene Expression Imputation

GatorST also enables imputation of gene expression values by projecting the learned embeddings back into the gene expression space. Here, imputation is applied as a post hoc step based on the learned embeddings, rather than being jointly optimized with the contrastive and classification objectives during the main training stage. A linear transformation is applied:

$$\;\hat{X}=W_{X}\;H+b_{X}.$$

### 4.8 Trajectory Inference

To reconstruct developmental or spatial trajectories, GatorST embeddings can be utilized as input for established trajectory inference tools such as PAGA [27]. Specifically, we applied the PAGA method to the embeddings derived from two representative DLPFC slices: #151675 and #151676. PAGA then constructs a connectivity graph where nodes correspond to discrete clusters (e.g., cortical layers), edges indicate transcriptional transitions between these clusters, and edge weights reflect the degree of transcriptomic continuity. This setup allows GatorST to support biologically plausible trajectory modeling. To enhance visualization, UMAP [26] is employed to project the learned embeddings into a two‐dimensional space, providing a clear and intuitive overview for subsequent trajectory reconstruction.

### 4.9 Batch Effect Removal

GatorST addresses batch effects by integrating Harmony into its framework. Harmony is applied to the latent embeddings across all 12 DLPFC tissue slices using the harmonypy (v0.0.9) implementation. The resulting batch‐corrected embeddings are used for both visualization (i.e., UMAP) and quantitative assessment via LISI metrics. LISI metrics were computed using the compute_lisi function provided in Harmonypy (v0.0.9), and two metrics were considered: cell‐type LISI (cLISI) and integration LISI (iLISI) [28]. cLISI evaluates local cell‐type diversity, where lower values indicate better preservation of biological structure. iLISI assesses mixing across datasets, with higher values reflecting superior batch integration. To facilitate comparison, both scores are normalized to the range [0, 1]:

$$n\;\left(cLISI\right)=\frac{N_{celltypes}-cLISI}{N_{celltypes}-1}\;,$$

$$n\;\left(iLISI\right)=\frac{iLISI-1}{N_{datasets}-1}\;,$$

where *N_celltypes_* is the number of clusters and *N_datasets_* is the number of datasets.

### 4.10 Benchmark Datasets

In this study, we assessed the performance of GatorST using various publicly available spatial transcriptomics datasets across different tissue types and spatial contexts. Specifically, we included the LIBD human dorsolateral prefrontal cortex (DLPFC) dataset [40], which consists of 12 tissue slices obtained using the 10x Visium platform; a human lymph node acquired from tissue containing germinal centers (GCs). We utilized two datasets from the 10 × Genomics Data Repository, including samples from human breast cancer and mouse brain tissue. Annotations for the human breast cancer and mouse brain anterior datasets are accessible from SEDR [23]. In addition to these Visium benchmarks, we incorporated three further datasets that span different tissues and technologies: an E9.5 mouse embryo dataset generated with Stereo‐seq, a Stereo‐seq dataset of mouse olfactory bulb, and a mouse hippocampus dataset profiled with Slide‐seqV2. These datasets include a wide range of gene expression profiles and spatial resolutions, providing a comprehensive benchmark for assessing the generalizability and robustness of the proposed method. Notably, no pre‐filtering was applied to the raw gene expression data. All available data were included in the analysis to ensure an unbiased and thorough evaluation.

### 4.11 Baseline Methods

To thoroughly assess the clustering performance of GatorST, we compared it with various state‐of‐the‐art spatial transcriptomics analysis methods. These included the Bayesian approach, BayesSpace, graph‐based models UTAG, Spatial‐MGCN, DeepST, GraphST, STAGATE, SpaGCN, and SpaceFlow, as well as contrastive learning‐based methods CCST and GraphST. Additionally, we compared GatorST with other representative methods, including BANKSY and spatially embedded deep representation (SEDR) [23]. BANKSY is a unified embedding framework that enhances gene expression profiles with features from spatial neighborhoods to jointly perform cell typing and domain segmentation. SEDR is a deep learning framework that integrates gene expression and spatial information by combining an autoencoder with a variational graph autoencoder. For all baselines that generate continuous latent embeddings, we applied a unified downstream clustering procedure using K‐means on the embeddings (with the number of clusters set to the dataset‐specific annotation level) to ensure fair comparison. BayesSpace and BANKSY were run with their intrinsic domain inference settings, as they do not rely on an external clustering step.

### 4.12 Performance Evaluation Metrics

We conducted a rigorous evaluation of the proposed method using five commonly used metrics for clustering performance, including the Adjusted Rand Index (ARI), Normalized Mutual Information (NMI), clustering accuracy (ACC), purity, and Homogeneity. Specifically, ARI measures the agreement between the predicted and reference clusterings while accounting for randomness. This makes ARI particularly useful for assessing the reliability of clustering, particularly in the presence of overlapping classes. NMI quantifies the mutual dependence between the predicted and actual labels. By normalizing mutual information, it accounts for label distribution imbalances, with higher scores suggesting stronger agreement. ACC reflects the proportion of correctly assigned instances. It is calculated by optimally aligning the predicted clusters with the ground truth. Purity evaluates how well each predicted cluster predominantly consists of members from a single ground truth class. It reflects the coherence within each cluster. Homogeneity measures the degree to which clusters consist entirely of data points from a single class. Higher values are better for all these clustering evaluation metrics.

For the data imputation task, we employed three commonly used evaluation metrics: the Pearson Correlation Coefficient (PCC), L1 loss, and Root Mean Square Error (RMSE) [41]. In particular, PCC measures the linear correlation between the imputed and ground truth values, with higher scores suggesting better preservation of global structural patterns in the data. The loss of L1 captures the average absolute deviation between predicted and actual values. Its robustness to outliers makes it a reliable metric for assessing local imputation accuracy. RMSE computes the square root of the mean squared error and emphasizes larger deviations due to its quadratic penalization. A higher PCC reflects better global similarity to the ground truth, while lower L1 loss and RMSE values reflect smaller prediction errors, suggesting more accurate imputations. These metrics provide a comprehensive assessment of both the global consistency and the local precision of the imputed results.

To ensure robustness and generalizability across different tissues and data distributions, GatorST employs a multi‐run cross‐validation approach. Specifically, for each dataset, the data loader (loader_construction) partitions samples into train, validation, and test sets using fixed proportions (80%/10%/10%). This process is repeated across 10 random seeds to assess consistency. Each run reinitializes the model and randomizes data splits, ensuring that results reflect average and variance across independent splits.

For each method and dataset, experiments were repeated 10 times with different random seeds, and the mean score across runs was used as the dataset‐level summary statistic. Repeated runs from the same dataset were not treated as independent observations in hypothesis testing, thereby avoiding pseudo‐replication. For Figure 2B, statistical significance was assessed using pairwise two‐sided Wilcoxon signed‐rank tests comparing GatorST with each baseline on the paired dataset‐level mean scores for each clustering metric across the 16 benchmark datasets. For Figure 3B, the same procedure was applied to PCC, L1 loss, and RMSE across the 18 benchmark datasets. The Wilcoxon signed‐rank test was chosen because all methods were evaluated on the same datasets, and no normality assumption was imposed on the metric distributions. This test assumes that observations are paired, that paired differences are independent across datasets, and that the distribution of paired differences is approximately symmetric around the median. Significance was denoted as n.s. (*p* ≥ 0.05), ^*^ (*p* < 0.05), ^**^ (*p* < 0.005), ^***^ (*p* < 0.0005), and ^****^ (*p* < 0.00005).

### 4.13 Code Availability

GatorST is provided as a Python package available at https://github.com/zhangzh1328/GatorST, with detailed functions for implementation.

## Funding

J.B. is supported by National Institutes of Health grants R01AG083039, RF1AG084178, RF1AG077820, R01AG080991, R01AG080624, and R01AG076234. Q.S. is supported by the National Institute of General Medical Sciences of the National Institutes of Health (R35GM151089).

## Conflicts of Interest

The authors declare no conflicts of interest.

## Data Availability

The spatial transcriptomics datasets analyzed in this study are publicly available from the following sources: the LIBD human dorsolateral prefrontal cortex (DLPFC) dataset, which was obtained using the 10x Visium platform (http://research.libd.org/spatialLIBD/); human lymph node Visium dataset acquired from tissue containing germinal centers (GCs) and obtained from GEO (accession no. GSE263617); the human breast cancer dataset (https://www.10xgenomics.com/datasets/human‐breast‐cancer‐block‐a‐section‐1‐1‐standard‐1‐1‐0) and the mouse brain tissue dataset (https://www.10xgenomics.com/datasets/mouse‐brain‐serial‐section‐1‐sagittal‐anterior‐1‐standard‐1‐1‐0), both obtained from the 10x Genomics Data Repository. In addition, we used an E9.5 mouse embryo dataset generated with Stereo‐seq and downloaded from the MOSTA resource (https://db.cngb.org/stomics/mosta/), a Stereo‐seq dataset of mouse olfactory bulb (https://github.com/JinmiaoChenLab/SEDR_analyses), and a mouse hippocampus dataset profiled with Slide‐seqV2 (https://portals.broadinstitute.org/single_cell/study/slide‐seq‐study).
